# Investigation of the Effect on the Albumin Binding Moiety for the Pharmacokinetic Properties of ^68^Ga-, ^205/206^Bi-, and ^177^Lu-Labeled NAPamide-Based Radiopharmaceuticals

**DOI:** 10.3390/ph16091280

**Published:** 2023-09-11

**Authors:** Dániel Szücs, Judit P. Szabó, Viktória Arató, Barbara Gyuricza, Dezső Szikra, Imre Tóth, Zita Képes, György Trencsényi, Anikó Fekete

**Affiliations:** 1 Division of Nuclear Medicine and Translational Imaging, Department of Medical Imaging, Faculty of Medicine, University of Debrecen, Nagyerdei krt. 98, H-4032 Debrecen, Hungary; szucs.daniel@science.unideb.hu (D.S.); szabo.judit@med.unideb.hu (J.P.S.); arato.viktoria@med.unideb.hu (V.A.); gyuricza.barbara@med.unideb.hu (B.G.); szikra.dezso@med.unideb.hu (D.S.); kepes.zita@med.unideb.hu (Z.K.); trencsenyi.gyorgy@med.unideb.hu (G.T.); 2 Department of Physical Chemistry, Faculty of Science and Technology, University of Debrecen, Egyetem tér 1, H-4032 Debrecen, Hungary; imre.toth@science.unideb.hu; 3 Doctoral School of Chemistry, Faculty of Science and Technology, University of Debrecen, Egyetem tér 1, H-4032 Debrecen, Hungary; 4 Doctoral School of Clinical Medicine, Faculty of Medicine, University of Debrecen, Nagyerdei krt. 98, H-4032 Debrecen, Hungary; 5 Doctoral School of Pharmaceutical Sciences, Faculty of Pharmacy, University of Debrecen, Nagyerdei krt. 98, H-4032 Debrecen, Hungary

**Keywords:** albumin-binding moiety, malignant melanoma (MM), melanocortin-1 receptor (MC1-R), NAPamide, positron emission tomography (PET), radiolabeling, single-photon emission computed tomography/computed tomography (SPECT/CT)

## Abstract

Although radiolabeled alpha-melanocyte stimulating hormone-analogue NAPamide derivatives are valuable melanoma-specific diagnostic probes, their rapid elimination kinetics and high renal uptake may preclude them from being used in clinical settings. We aimed at improving the pharmacokinetics of radiolabeled DOTA-NAPamide compounds by incorporating a 4-(p-iodo-phenyl)-butanoic acid (IPB) into the molecules. Followed by ^68^Ga-, ^205/206^Bi-, and ^177^Lu-labelling, the radiopharmaceuticals ([^68^Ga]Ga-DOTA-IPB-NAPamide, [^205/206^Bi]Bi-DOTA-IPB-NAPamide, [^177^Lu]Lu-DOTA-IPB-NAPamide) were characterized in vitro. To test the imaging behavior of the IPB-containing probes, B16F10 tumor-bearing C57BL/6 mice were subjected to in vivo microPET/microSPECT/CT imaging and ex vivo biodistribution studies. All tracers were stable in vitro, with radiochemical purity exceeding 98%. The use of albumin-binding moiety lengthened the in vivo biological half-life of the IPB-carrying radiopharmaceuticals, resulting in elevated tumor accumulation. Both [^68^Ga]Ga-DOTA-IPB-NAPamide (5.06 ± 1.08 %ID/g) and [^205/206^Bi]Bi-DOTA-IPB-NAPamide (4.50 ± 0.98 %ID/g) exhibited higher B16F10 tumor concentrations than their matches without the albumin-binding residue ([^68^Ga]Ga-DOTA-NAPamide and [^205/206^Bi]Bi-DOTA-NAPamide: 1.18 ± 0.27 %ID/g and 3.14 ± 0.32; respectively), however; the large amounts of off-target radioactivity do not confirm the benefits of half-life extension for short-lived isotopes. Enhanced [^177^Lu]Lu-DOTA-IPB-NAPamide tumor uptake even 24 h post-injection proved the advantage of IPB-based prolonged circulation time regarding long-lived radionuclides, although the significant background noise must be addressed in this case as well.

## 1. Introduction

Malignant melanoma (MM) derived from melanocytes is the fifth most common cancer among adults in the United States. In addition, being a highly aggressive neoplasm, MM is responsible for 75% of deaths caused by skin cancer [1]. Therefore, the development of effective melanoma specific diagnostic and therapeutic vectors is of paramount importance to increase the survival rate of patients. Besides providing an essential contribution to the growth of melanoma cells, the melanocortin type 1 receptor (MC1-R)—also named melanocyte-stimulating hormone receptor (MSHR)—is abundantly expressed on the surface of melanin positive primary MM and related metastatic lesions [2]. Consequently, MC1-R seems to be a useful target for selective melanoma imaging and therapy. Beyond being stimulated by α-Melanocyte-stimulating hormone (α-MSH), the MC1-R receptor can also exert its effects via activation by the specific binding of various α-MSH analogues [3]. Previous studies proved that radiolabeled α-MSH analogue NAPamide (Ac-Nle-Asp-His-D-Phe-Arg-Trp-Gly-Lys) derivatives serve as effective molecular probes for the determination of the MC1-R expression pattern of the melanoma cells [4,5]. Although the existing radiolabeled NAPamide analogues are characterized by relatively high tumor uptake, their rapid elimination kinetics along with the high renal uptake must be addressed [6].

As peptides exert meaningful binding affinity and specificity to various tumor biomarkers, coupling them with radioisotopes to transport cytotoxic radiation to tumor cells appears to be a valuable strategy for both imaging and therapeutic purposes [7]. From a clinical point of view, the labelling of the chelator–target molecule conjugate with both diagnostic and therapeutic radionuclides has its clear advantages. For this purpose, the application of chelator DOTA (1,4,7,10-tetraazacyclododecane-1,4,7,10-tetraacetic acid) is appropriate because it forms a stable complex with different diagnostic and therapeutic radiometals including gallium-68 (^68^Ga), lutetium-177 (^177^Lu) and bismuth-213 (^213^Bi).

The short biological half-life, rapid clearance and related lower tumor uptake and retention, as well as the high renal uptake of peptide-based radiocomplexes, constitute major drawbacks for both PET imaging and therapeutic applications [8]. To overcome these shortcomings, the optimization of the structure of the radioligands is often required for their clinical usage regarding the linker unit between the chelator and vector molecules, which affects the pharmacokinetic properties of the labeled compounds. Therefore, the modification of the linker may improve the diagnostic and the therapeutic potential of the radiopharmaceutical. The introduction of an albumin-binding moiety is an increasingly investigated method for the prolongation of the blood circulation time of the radiolabeled peptides, and subsequent increase in tumor accumulation and a decrease in renal uptake. In addition, using an albumin-binding unit containing radiopharmaceuticals, a smaller amount of radioactivity is sufficient for successful therapy compared to their unmodified counterparts. Hence, the presence of the albumin-binding motif has a meaningful effect on the tumor-targeting capability and the pharmacokinetic profile of the radioligands, that are crucial for the achievement of improved image quality and effective targeted radiation therapy as well. In addition, some studies revealed the albumin-binding ability of the 4-(p-iodophenyl) butyric acid—discovered from a DNA-encoded chemical library—and its successful use for the lengthening of the circulation time of folate—and prostate specific membrane antigen (PSMA)-based radiopharmaceuticals [9,10,11].

During targeted radionuclide therapy (TRNT), it is of utmost importance that the cytotoxic radiation reaches the cancer cells as specifically as possible without exerting adverse toxic effects on the surrounding healthy tissues. Thus, to achieve successful TRNT, data derived from biodistribution studies could be used for the determination of the localization and the retention of the radiopharmaceutical in the targeted organs and tissues. Although β-particle emitting radionuclides—for example, ^177^Lu isotope—are applied most often in TRNT settings, their low linear energy transfer (LET 0.2 keV/μm) value as well as long path in tissue (1–5 mm) lead to the destruction of healthy cells and hamper their widespread clinical usage [12]. Given the short-range (50–100 µm) and high LET (50–230 keV/μm) of α-emitting radionuclides such as ^213^Bi (t_1/2_ = 46 min, alpha energy of 8.4 MeV, tissue penetration: 85 μm) that ensure a selective tumor cell killing effect sparing the nearby intact tissues, targeted alpha therapy (TAT) has become the focus of increasing scientific and clinical interest [13].

Based on the considerations mentioned above, we designed the synthesis and performed the preclinical evaluation of ^68^Ga-, ^177^Lu- and ^213^Bi-labeled, DOTA-conjugated NAPamide radioligands containing 4-(p-iodophenyl)butyryl group (IPB) as an albumin binding moiety in order to investigate their diagnostic and therapeutic feasibility.

## 2. Results and Discussion

As for metal-based tracers, the radiometal and the targeting vector are combined with the chelating agent and the linker in an inert and stable complex. The usefulness of the integration of an albumin-binding unit in the reduction of the rapid clearance of ^177^Lu-labeled radiofolate was first realized by Müller et al. [9] who used 4-(p-iodophenyl)butyryl moiety (IPB) as an albumin-binder. Later, this method was applied to reduce the renal and the hepatic accumulation of some PSMA-based radiopharmaceuticals using different albumin binding moieties [10,11]. Currently, we aimed at incorporating the IPB motif into MM targeting and radiolabeled the DOTA-NAPamide compound to reduce its rapid elimination and high renal uptake.

### 2.1. Synthesis of DOTA-NAPamide Containing 4-(p-iodo-phenyl)butyryl Group (***10***)

**Scheme 1:**
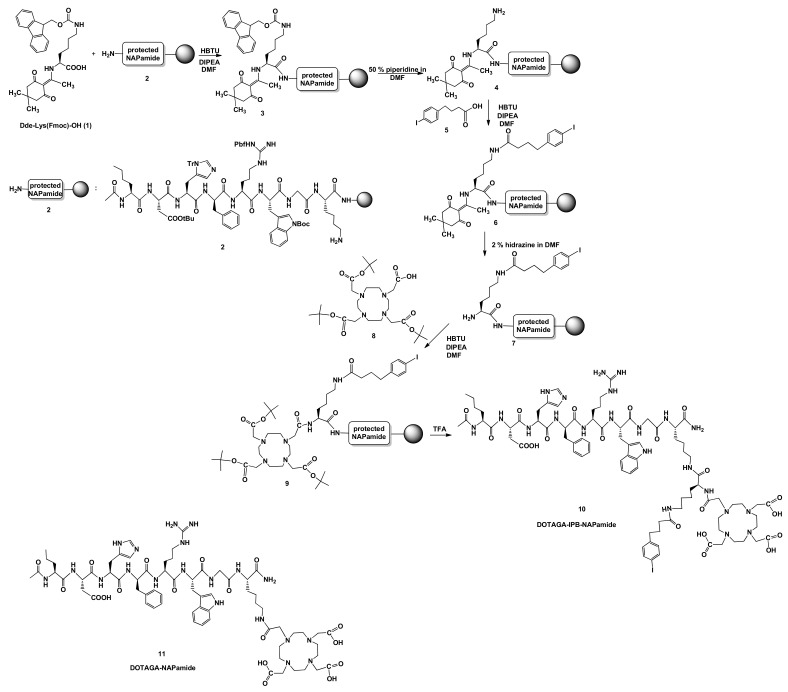
Synthesis of DOTA-IPB-NAPamide (**10**).

The designed NAPamide derivative **10** was prepared by multistep solid-phase peptide synthesis (as seen on Scheme 1) using a method similar to that described by Umbrict et al. [11]. In the first step, a fluorenylmethoxycarbonyl (Fmoc)- and 1-(4,4-dimethyl-2,6-dioxocyclohex-1-ylidene)ethyl (Dde)-protected lysine (**1**) was reacted with a commercially available and selectively protected NAPamide molecule **2**—immobilized to resin—using *O*-(benzotriazol-1-yl)-*N,N,N*′,*N*′-tetramethyluronium hexafluorophosphate (HBTU) as a coupling agent in the presence ethyldiisopropylamine (DIPEA) in *N,N*-dimethylformamide (DMF). After deprotection, 4-(p-iodophenyl)butanoic acid (**5**) was conjugated to the NAPamide derivative **4** applying the previously mentioned coupling method. Then, the Dde protecting group was removed with a solution of 2% hydrazine in DMF. Afterwards, DOTA-tris(t-Bu)ester (**8**) was attached to the free amino group of the peptide **7**. Finally, the protective groups were hydrolyzed and the NAPamide derivative was cleaved from the resin by treatment with trifluoroacetic acid (TFA) to yield the 4-(p-iodophenyl)butyryl group containing DOTA-NAPamide molecule (**10**). The compound **10** was purified using semipreparative HPLC and characterized by HR-MS.

### 2.2. Radiochemistry

Based on their decay properties, radiometals can be used for both diagnostic and therapeutic purposes. The radiation emitted by diagnostic radiometals interacts weakly with the examined tissues, and is able to reach the external detector. On the contrary, however, radioisotopes with therapeutic rays emit locally cytotoxic radiation.

**Figure 1:**
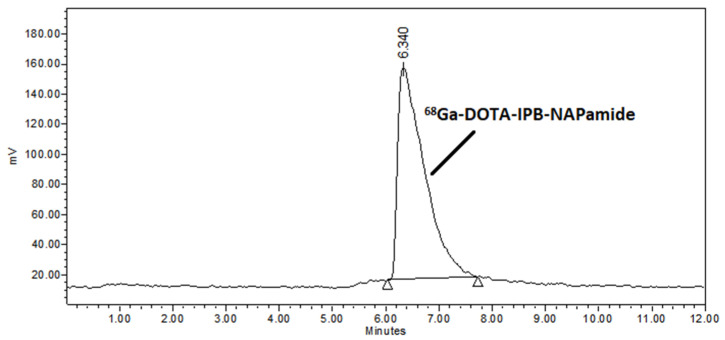
Radio-HPLC chromatogram of ^68^Ga-labeled radiopharmaceutical.

**Figure 2:**
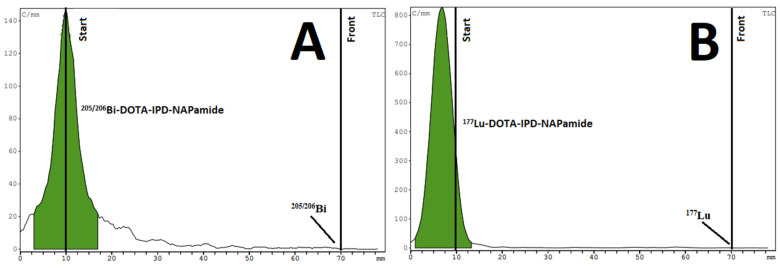
(**A**) Radio-iTLC chromatogram of ^205/206^Bi-labeled radiopharmaceutical; (**B**) Radio-iTLC chromatogram of ^177^Lu-labeled radiotracer. Eluens: 0.5 M citric acid pH 5.5.

^68^Ga-labeled peptides seem to be strong applicants for the PET-based detection of different types of tumors providing an auspicious opportunity to plan an effective therapeutic strategy and to monitor the therapeutic response. Therefore, we first performed the radiolabeling of DOTA-IPB-NAPamide (**10**) using diagnostic ^68^Ga (*t*_1/2_ = 68 min, I = 89%, *E_max_(β^+^)* = 1.92 MeV) that was produced in a cyclotron via ^68^Zn(p,n)^68^Ga nuclear reaction with a high labeling yield (>98%). For the accomplishment of preliminary preclinical studies, compound **10** was labeled with ^205/206^Bi isotope—a surrogate of alpha-emitting ^213^Bi isotope. Identically to the incorporation of [^68^Ga]Ga^3+^, high labeling yield (>98%) was experienced in case of the ^205/206^Bi isotopes. Finally, the radiolabeling of precursor **10** was carried out with beta-emitting, long-lived ^177^Lu isotope (*t*_1/2_ = 6.647 days). Furthermore, we conducted the radiolabeling of the commercially available DOTA-NAPamide with ^68^Ga, ^205/206^Bi and ^177^Lu radiometals and used these radiopharmaceuticals as references. The labeled complexes were purified by solid-phase extraction on a Sep-Pak C18 Plus Light column. The ^68^Ga-labeled radiopharmaceuticals were analyzed using radio-HPLC (Figure 1), while radio instant thin-layer chromatography (iTLC) was applied for the characterization of the ^205/206^Bi- and ^177^Lu-labeled complexes (Figure 2). In all cases, the radiochemical purity of the radiotracers exceeded 98%.

The stability test of the purified labeled derivatives was performed in the presence of rat serum, 0.01 mM EDTA solution and metal ions (Mg^2+^: 0.51 mM, Ca^2+^: 1.14 mM, Zn^2+^: 0.01 mM, Cu^2+^: 0.001 mM) separately. After a 2 h incubation, the radiocomplexes showed high stability in the serum and inertness towards transchelation and transmethylation under the examined conditions. The stability of the ^177^Lu-labeled complexes was investigated over 2 days.

Furthermore, the *logP* values of the labeled compounds were determined, with the respective values being −3.46, −2.12, −3.65, −2.32, −2.6, and −1,26 for [^68^Ga]Ga-DOTA-NAPamide, [^68^Ga]Ga-DOTA-IPB-NAPamide, [^205/206^Bi]Bi-DOTA-NAPamide, [^205/206^Bi]Bi-DOTA-IPB-NAPamide, [^177^Lu]Lu-DOTA-NAPamide, and [^177^Lu]Lu-DOTA-IPB-NAPamide. Supported by the *logP* values, the albumin binding unit (IPB) containing complexes proved to be more lipophilic than the counterparts lacking the IPB motif that results in the reduced renal uptake along with elevated hepatic accumulation of the tracers with the IPB sequence.

### 2.3. Biology

#### 2.3.1. In Vivo and Ex Vivo Biodistribution of ^68^Ga-Labeled DOTA-NAPamide and DOTA-IPB-NAPamide

To assess the tumor targeting potential and the organ distribution of [^68^Ga]Ga-DOTA-NAPamide and [^68^Ga]Ga-DOTA-IPB-NAPamide, MC1-R overexpressing B16F10 melanoma tumor-bearing mice were subjected to miniPET examination and post imaging uptake studies. It is well established from the literature that the B16F10 tumor of mouse origin is a competent model for the research and the development of radiopharmaceuticals targeting malignant melanoma due to the fact that it exhibits high MC1-R expression with more than 20,000 MC1-R per melanoma cell [14].

**Figure 3:**
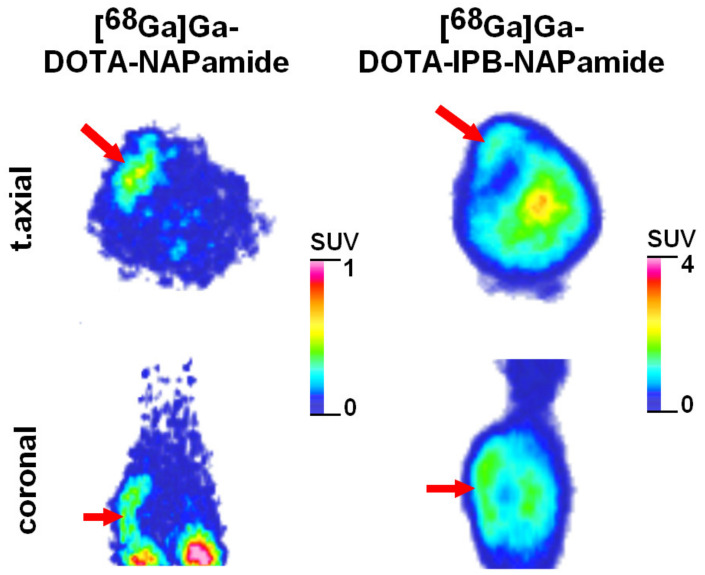
Representative static PET images of B16F10 melanoma xenografted mice 90 min post administration of [^68^Ga]Ga-DOTA-NAPamide and [^68^Ga]Ga-DOTA-IPB-NAPamide and 9 ± 1 days following MC1-R positive mouse B16F10 melanoma cell implantation. Red arrows: B16F10 tumor. PET: positron emission tomography; MC1-R: melanocortin-1 receptor.

Upon qualitative PET image assessment, the *sc.* growing tumors were unequivocally identifiable using both MC1-R specific radiopharmaceuticals; however, higher background-activity was observed for [^68^Ga]Ga-DOTA-IPB-NAPamide (as displayed in Figure 3). The sufficient tumor accumulation of the ^68^Ga-labeled probes strengthened their MC1-R binding ability as well as their tumor-homing potential. This was consistent with the findings of Nagy et al., who tested the MC1-R selectivity of DOTA chelated NAPamide molecules labeled with ^68^Ga and ^44^Sc in receptor positive B16F10 cells and A375 cell lines with low receptor expression, and pointed out that the tracer accumulation was notably higher in the B16F10 cells with high receptor density relative to the A375 cells without significant MC1-R presence (*p* < 0.01) [15]. Corresponding to our results, Cheng et al. and Tafreshi et al. also registered increased uptake of radiolabeled α-MSH analogues in B16F10 mouse melanoma cells [14,16,17].

Based on the evaluation of the decay-corrected PET scans, a notable difference was encountered between the two ^68^Ga-labeled probes with respective SUV_mean_ values being 0.43 ± 0.09 and 1.31 ± 0.24 for [^68^Ga]Ga-DOTA-NAPamide and [^68^Ga]Ga-DOTA-IPB-NAPamide (*p* ≤ 0.05). This is in line with previous literature data, according to which using different albumin-binding unit such as 4-(p-iodo-phenyl)-butanoic acid moiety (IPB) to lengthen the half-life of small, tumor-specific molecules (for example PSMA) results in elevated uptake in malignant lesions [18,19].

In agreement with the in vivo data, the increased circulation time was also reflected in the results of the post imaging biodistribution studies. Using [^68^Ga]Ga-DOTA-IPB-NAPamide, notably higher radiotracer accretion was registered in most of the selected organs and tissues compared with that of [^68^Ga]Ga-DOTA-NAPamide lacking in the albumin-binding motif (*p* ≤ 0.01; seen in Table 1). Besides the presence of the albumin-binding unit, however, the amount of the injected peptide and related saturation of the receptor binding sites as well as the distinct activity of the radiopeptide could also exert a meaningful impact on the uptake pattern [20,21]. The prolonged circulation time of [^68^Ga]Ga-DOTA-IPB-NAPamide was further validated by its prominently high activity in the blood (%ID/g: 14.21 ± 1.87 at 90 min post injection). In addition, the currently presented ex vivo %ID/g data as well as the in vivo SUV_mean_ values of [^68^Ga]Ga-DOTA-NAPamide correlated well with previous studies, where the distribution and the tumor homing properties of receptor-affine [^68^Ga]Ga-DOTA-NAPamide and [^44^Sc]Sc-DOTA-NAPamide probes were assessed in B16F10 experimental tumor model, and identical organ distribution pattern and tumor uptake were observed [15].

Upon the evaluation of the uptake pattern of [^68^Ga]Ga-DOTA-NAPamide—except for the kidneys—faint radioactivity was detected in the investigated abdominal and thoracic organs. Consistent results were reported in earlier works with ^111^In-, ^67^Ga- and ^68^Ga-labeled DOTA-NAPamide compounds that also demonstrated negligible abdominal and thoracic activity [15,20,22,23]. On the contrary, however, Cheng et al. found considerable uptake of ^18^F- and ^64^Cu-labeled NAPamide in the majority of the abdominal and thoracic organs including the lungs, the heart, the stomach, the intestines, the spleen, the pancreas as well as the liver [14,16].

#### 2.3.2. Ex Vivo Biodistribution of [^205/206^Bi]Bi-DOTA-NAPamide and [^205/206^Bi]Bi-DOTA-IPB-NAPamide

For the assessment of the MC1-R specificity and the tissue uptake pattern of [^205/206^Bi]Bi-DOTA-NAPamide and [^205/206^Bi]Bi-DOTA-IPB-NAPamide, ex vivo biodistribution studies were performed applying C57BL/6 male mice bearing B16F10 tumors. The %ID/g data acquired from the ^205/206^Bi-based experiments were in strong correlation with the uptake values of the ^68^Ga-labeled PET diagnostic probes (demonstrated in Table 2). Comparing the two ^205/206^Bi-labeled tracers, the radiopharmaceutical concentration of the evaluated organs and tissues was markedly higher (*p* ≤ 0.05 and *p* ≤ 0.01) upon [^205/206^Bi]Bi-DOTA-IPB-NAPamide administration than in the case of [^205/206^Bi]Bi-DOTA-NAPamide. Due to its lipophilic property—that was in line with the LogP value—the albumin moiety containing compound displayed higher hepatic and lower renal accumulation (presented in Table 2). This indicated that the elimination of the [^205/206^Bi]Bi-DOTA-IPB-NAPamide occurred mainly via the hepatobiliary system. Moreover, the elevated IPB-NAPamide uptake of the liver was in correlation with results of previous experiments dealing with ^18^F-labeled NAPamide and ^213^Bi-labeled FOLDamide compounds [14,24].

In addition, different biodistribution profiles between the two ^213^Bi-labeled probes were observed specifically in the blood, and in organs with congestion (e.g., lung, heart) with higher radiotracer concentration in the [^205/206^Bi]Bi-DOTA-IPB-NAPamide administered group that further strengthened the increased circulatory half-life of the radiopharmaceutical induced by the albumin-binding sequence. Upon the assessment of the uptake of [^205/206^Bi]Bi-DOTA-NAPamide without the IPB motif, a moderate radiotracer concentration was depicted in all organs and tissues, which was in agreement with earlier research findings on ^213^Bi-labeled NAPamide compound [24]. A rapid renal clearance of [^205/206^Bi]Bi-DOTA-NAPamide was evidenced by the low blood activities and the obvious kidney uptake (8.78 ± 3.61 %ID/g).

Within the framework of the ex vivo organ distribution experiments, 9 ± 1 days post B16F10 cell implantation, the MC1-R selectivity of the ^205/206^Bi-labeled DOTA conjugated NAPamide derivatives was evaluated in the corresponding melanoma tumor-bearing mice 90 minutes after the *iv.* injection of 2.39 ± 0.19 MBq of [^205/206^Bi]Bi-DOTA-NAPamide or [^205/206^Bi]Bi-DOTA-IPB-NAPamide. The high radiotracer uptake of the B16F10 tumors confirmed the tumor-targeting ability of the ^205/206^Bi-labeled NAPamide molecules (3.14 ± 0.32 and 4.50 ± 0.98 for [^205/206^Bi]Bi-DOTA-NAPamide and [^205/206^Bi]Bi-DOTA-IPB-NAPamide, respectively). High tumor accumulation of [^205/206^Bi]Bi-DOTA-NAPamide coupled with much lower hepatic activity (%ID/g: 1.09 ± 0.17) and negligible uptake in the other assessed organs and tissues—apart from the kidneys—leads to elevated tumor-to-non-target ratios that allows for the acquisition of high contrasted images. In a similar manner, Kálmán-Szabó et al. [24] has recently published comparable ex vivo %ID/g data assessing the biodistribution and the MC1-R overexpressing tumor homing potential of a NAPamide-based radiotracer labeled with another isotope of bismuth, the alpha emitter ^213^Bi (^213^Bi]Bi-DOTA-NAPamide). Applying MC1-R positive melanoma tumor models, Kálmán-Szabó and co-workers reported elevated [^213^Bi]Bi-DOTA-NAPamide uptake in the B16F10 tumors 90 min post administration (%ID/g: 3.76 ± 0.96). Apart from [^213^Bi]Bi-DOTA-NAPamide, they also proposed the synthesis and the radiolabeling of the following novel MC1-R selective amide derivatives: HOLDamide, FOLDamide and MARSamide [24]. Similarly to the NAPamide molecule, the other probes also seemed to be feasible in the imaging of receptor expressing B16F10 tumors. Although the neoplasms could be visualized using all investigated tracers, the highest radioactivities were recorded in case of the ^213^Bi-labeled NAPamide and FOLDamide compounds with respective accumulation figures being 3.76 ± 0.96 %ID/g and 3.28 ± 0.95 %ID/g.

#### 2.3.3. In Vivo and Ex Vivo Biodistribution Pattern of [^177^Lu]Lu-DOTA-NAPamide and [^177^Lu]Lu-DOTA-IPB-NAPamide

**Figure 4:**
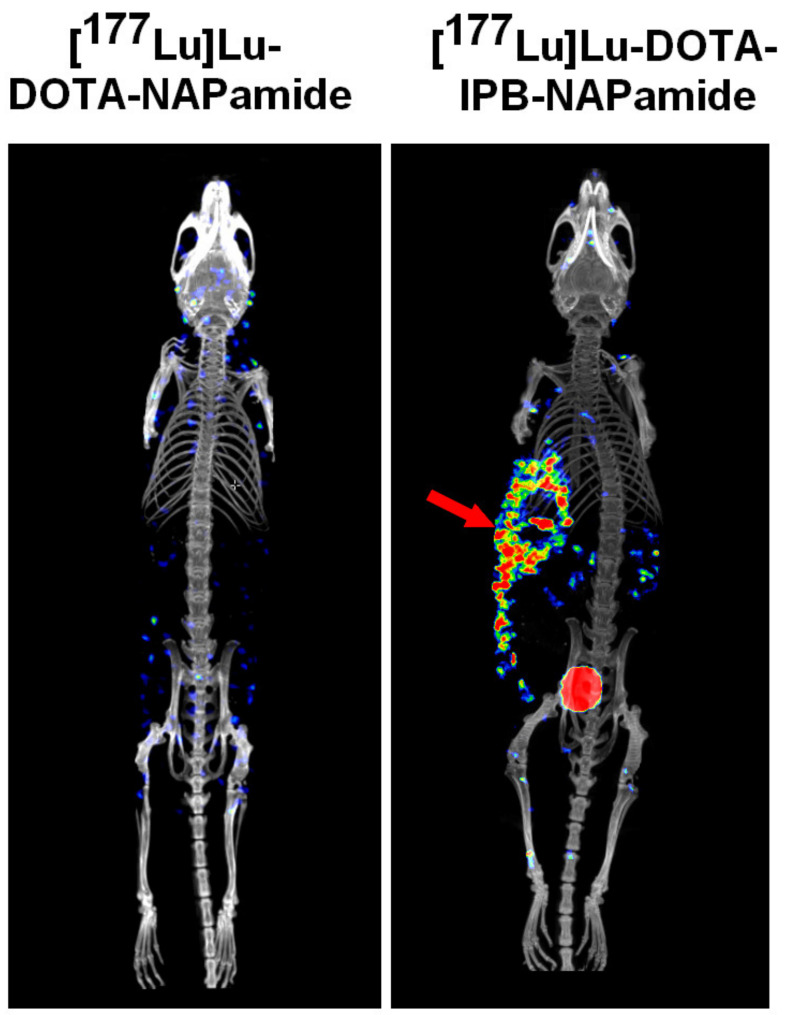
Representative MIP SPECT/CT scans of B16F10 tumorous mice 24 h post intravenous administration of [^177^Lu]Lu-DOTA-NAPamide and [^177^Lu]Lu-DOTA-IPB-NAPamide and 9 ± 1 days after subcutaneous MC1-R positive B16F10 cell implantation. Red arrow: B16F10 tumor. MIP: 3D Maximum Intensity Projection; SPECT/CT: Single Photon Emission Computed Tomography/Computed Tomography.

In the last part of our experiments, in vivo imaging and post imaging organ distribution experiments were performed for the determination of the uptake pattern and the tumor targeting potential of MC1-R specific, β-emitter ^177^Lu-labeled NAPamide probes either in the presence or in the absence of the IPB albumin-binding sequence ([^177^Lu]Lu-DOTA-NAPamide and [^177^Lu]Lu-DOTA-IPB-NAPamide). The in vivo SPECT/CT imaging studies revealed that the *sc.* growing B16F10 tumors could be clearly identified 24 h post injection of [^177^Lu]Lu-DOTA-IPB-NAPamide; however, using the radiopharmaceutical without the albumin-binding IPB, the malignancy was not detectable at the same investigation time point (Figure 4).

This was in accordance with the ex vivo figures that also confirmed significantly more elevated (*p* ≤ 0.01) tumor uptake of [^177^Lu]Lu-DOTA-IPB-NAPamide in comparison with [^177^Lu]Lu-DOTA-NAPamide. Identically to the results with the ^68^Ga- and the ^205/206^Bi-labeled counterparts, the incorporation of the albumin binding moiety could be responsible for the more increased tumor uptake as well as tumor retention. In correlation with the results on the ^68^Ga-labeled probes (Table 1), and in accordance with the *LogP* values, higher liver accumulation was observed for the IPB-containing ^177^Lu-labeled radiopharmaceutical; however, interestingly, the kidney activity was also elevated (Table 3). This may indicate that the probe is cleared through the kidneys as well as the hepatobiliary system. Comparing the distribution data of the currently assessed ^177^Lu-labeled molecules with the observations of other research studies, we also found that the kidney activity was the highest 24 h post injection of the radiopharmaceuticals. Similarly to our result, Guo and Miao—who explored the post-imaging distribution of MC1-R specific ^177^Lu-DOTA-GGNle-CycMSHhex radiopharmaceutical using a B16/F1 tumor model—also reported meaningful tumor accumulation (8.24 ± 1.51 %ID) one day post tracer administration; however, faint blood activity (0.23 ± 0.41 %ID) was registered at the same time point, which contradicted our observations [25]. In addition, the relatively high renal ^177^Lu-DOTA-GGNle-CycMSHhex accretion (4.75 ± 1.03 %ID) 24 h post tracer injection was in line with the current results [25].

The ex vivo biodistribution data of [^177^Lu]Lu-DOTA-NAPamide and [^177^Lu]Lu-DOTA-IPB-NAPamide are displayed in Table 3.

Summarizing the in vivo imaging and ex vivo biological findings, we can draw the conclusion that the albumin-binding sequence containing ^68^Ga-, ^205/206^Bi- and ^177^Lu-labeled MC1-R specific NAPamide radiopharmaceuticals compared with the ones without the IPB motif exhibited significantly higher accumulation in the examined organs and in the B16F10 tumors as well due to their increased circulation time. Given the selective MC1-R binding ability of all assessed probes—regardless of the imaging label—these radiolabeled, amide-based derivatives could serve as a magic bullet for the in vivo molecular diagnostics of MCR-1-overexpressing malignant melanoma. Finally, the NAPamide-based molecules labeled with α-emitter ^213^Bi or β-emitter ^177^Lu could play a pivotal role in the targeted radiotherapy of MC1-R-positive cancers. Therefore, these radiopharmaceuticals display meaningful potential for theranostic applications as well.

## 3. Materials and Methods

### 3.1. General

DOTA was purchased from ChemaTech (Dijon, France). DOTA-NAPamide was from ABX (Radeberg, Germany), while Ac-Nle-Asp(tBu)-His(Trt)-(D-Phe)-Arg(Pbf)-Trp(Boc)-Gly-Lys-(rink Amide MBHA resin) (resin-bound NAPamide (**2**)) was acquired from CASLO (Lyngby, Denmark). All other reagents were obtained from Sigma-Aldrich (Budapest, Hungary). For LC-MS, a Waters Acquity UPLC Iclass system was applied with HPLC-MS grade acetonitrile (ACN), MeOH (Fisher Solutions, El Cajon, CA, USA) and deionized water (Milli-Q, 18.2 MΩcm^−1^, Merck, Kenilworth, NJ, USA). Cyclotron-produced ^68^Ga and ^205/206^Bi radioisotopes were obtained from GE PETtrace cyclotron at the Division of Nuclear Medicine, Department of Medical Imaging, University of Debrecen, Debrecen, Hungary. ^177^Lu was purchased from IZINTA Trading Co. Ltd., (Budapest, Hungary). Radioactivity of the samples was determined by CAPINTEC CRC-15PET dose calibrator and a Perkin Elmer Packard Cobra gamma counter (Llantirsant, UK). For semipreparative HPLC, a Waters LC Module 1 HPLC (Waters, Milford, CT, USA) was used with a Luna C18 10 µm (250 × 10 mm, Phenomenex, Torrance, CA, USA) column (eluent A: 0.01% TFA, eluent B: ACN). While for analytical HPLC and radio-HPLC, a Waters 2695 Alliance HPLC system (Waters, Milford, MA, USA) was applied with a Luna C18 3 µm (150 × 4.6 mm, Phenomenex, Torrance, CA, USA) column (eluent A: 0.01% TFA, eluent B: 95% ACN). Both HPLC systems utilized an UV detector and an ATOMKI CsI scintillation detector. For radiochemical purification, Sep-Pak C18 Plus Light cartridges (Waters, Milford, MA, USA), and self-loaded TK-200 resin (100–150 μm, 70 mg, TrisKem, Bruz, France) were applied.

### 3.2. Chemistry

#### Synthesis of DOTA-IPB-NAPamide (**10**)

The synthesis of DOTA-IPB-NAPamide (**10**) was carried out similarly to the method described by Umbrict and et al. [11]. First, Dde-Lys(Fmoc)-OH (**1**) (38.3 mg, 0.072 mmol) was conjugated to resin-bound NAPamide (**2**) (0.018 mmol) with HBTU (27 mg, 0.071 mmol) and DIPEA (12.5 µL, 0.072 mmol) in anhydrous DMF (2 mL). The Fmoc group was cleaved with 50% piperidine in DMF (2 mL). Then, 4-(p-iodophenyl)butanoic acid (**5**) (35 mg, 0.072 mmol) was attached to the NAPamide derivative **4** in the presence of HBTU (27 mg, 0.071 mmol) and DIPEA, (12.5 µL, 0.072 mmol) in anhydrous DMF (2 mL). Afterwards, the Dde protecting group was hydrolyzed with 2 mL of 2% hydrazine in DMF. Subsequently, DOTA-tris(t-Bu)ester (**8**) (41 mg, 0.072 mmol) was coupled with the free amino group of the peptide **7**. Finally, the protective groups were hydrolyzed and the NAPamide derivative was cleaved from the resin with TFA (2 mL). After evaporation, the crude DOTA-IPB-NAPamide (**10**) was purified with semipreparative HPLC applying Luna C18 column (10 µm, 250 × 10 mm) with the following elution system: eluent A: 0.01% trifluoroacetic acid, eluent B: ACN, gradient: 0 min: 100% eluent A, 2 min: 100% eluent A, 32 min: 100% eluent B, 40 min: 100% eluent B 40.1 min: 100% eluent A at a flow rate of 4 mL/min. The t_R_ was 17.5 min, and the collected fraction was lyophilized to yield 11.7 mg of DOTA-IPB-NAPamide (34.5%) (Supplementary Material Figure S1). HR-MS (ESI, positive) *m*/*z* found: 944.4180 [M + H]^2+^, 629.6163 [M + H]^3+^, 472.7122 [M + H]^4+^; calculated: 944.4193 [M + H]^2+^ and 629.6145 [M + H]^3+^, 472.7136 [M + H]^4+^ (as seen in Supplementary Material Figure S2).

### 3.3. Radiochemistry

#### 3.3.1. ^68^Ga-Labeling of DOTA-NAPamide and DOTA-IPB-NAPamide (**10**)

^68^Ga nuclide was obtained from a cyclotron via ^68^Zn(p, n)^68^Ga nuclear reaction by the 10 min proton irradiation (12 MeV, 50 μA) of the pressed zinc disc (20 mg) to yield ~3 GBq ^68^Ga. Then, the Zn target was dissolved in hydrochloric acid (5 M, 5 mL), and the solution was passed through a ZR resin, then washed with 5 mL of hydrochloric acid (5 M). After elution of ^68^Ga with 5 mL of hydrochloric acid (2 M) the resulting solution was transferred into TK200 resin, which was also washed with 5 mL of hydrochloric acid (2 M). Then, ^68^Ga was fractionally eluted with hydrochloric acid (0.05 M). To this [^68^Ga]GaCl_3_ solution (400 μL, 130–200 MBq), 400 μL of NH_4_OAc (pH 4, 3 M), either 20 μL of DOTA-NAPamide stock solution (0.0125 µmol, 1 mg/mL) or 20 μL DOTA-IPB-NAPamide stock solution (0.0106 µmol, 1 mg/mL) were added. The reaction mixtures were heated at 95 °C for 15 min. Sep-Pak C18 Plus Light cartridge preconditioned with ethanol (5 mL) and water (5 mL) was applied for purification of radiolabeled derivatives. Water (1 mL) was used for the washing of the cartridge, while the elution of the radiolabeled ligands was carried out with 150 μL of 96% ethanol. The obtained solution was evaporated, and the residue was dissolved in water (300 µL). HPLC analysis was carried out with a Luna C18 3 µm (150 × 4.6 mm) column using the following gradient system: eluent A: 0.01% TFA and eluent B: 95% ACN; gradient: 0 min: 100% eluent A, 1 min: 100% eluent A, 10 min: 100% eluent B, 11 min: 100% eluent B, 12 min: 100% A at a flow rate of 1 mL/min. The radiochemical purity was investigated by radio-HPLC, eluent: 0.5 M citric acid pH 5.5.

#### 3.3.2. ^205/206^Bi-Labeling of DOTA-NAPamide and DOTA-IPB-NAPamide (**10**)

The ^205/206^Bi was obtained with a cyclotron using 60 min proton irradiation (12 MeV) on a natural Pb-foil target. Then, the Pb target was dissolved in 2 mL of 7 M nitric acid and evaporated to ~1 mL, and separated from the precipitation. Finally, 9 mL of water was added to and purified on a TK 200 resin self-filled column (70 mg) washed with 1 mL of nitric acid (0.7 M), 1 mL of nitric acid (7 M), and 5 mL of nitric acid (0.7 M) before use. After passing through the solution of ^205/206^Bi, the cartridge was washed with 5 mL of nitric acid (0.7 M), then the radionuclides were eluted with 2 mL of nitric acid (7 M). This ^205/206^Bi (~40 MBq) solution was concentrated and the residue was dissolved in 300 μL of 0.1 M hydrochloric acid. The ^205/206^Bi in hydrochloric acid (~20 MBq, 0.1 M, 150 µL), 3 M NH_4_OAc (pH 7, 450 µL), either 1 mg/mL DOTA-NAPamide aq. solution (0.0313 µmol 50 µL) or 1 mg/mL DOTA-IPB-NAPamide aq. solution (1 mg/mL, 0.0265 µmol, 50 µL), were mixed and incubated at 95 °C for 10 min. Then, the radioligands were purified on Sep-Pak C18 Plus Light cartridge washed with ethanol (5 mL) and water (5 mL) before being used. After loading of the reaction mixture, the column was washed with water (1 mL), and the radiolabeled compound was eluted with 96% ethanol (200 µL) and evaporated. Finally, the radiotracers were redissolved in 300 µL of water and its radiochemical purity was analyzed with radio-iTLC developed with 0.5 M citric acid (pH 5.5) as the mobile phase.

#### 3.3.3. ^177^Lu-Labeling of DOTA-NAPamide and DOTA-IPB-NAPamide (**10**)

[^177^Lu]LuCl_3_ (300 µL, 100–130 MBq) in 0.05 M HCl was mixed with 3 M NaOAc (300 µL, pH 4) either 90 µL of DOTA-NAPamide stock solution (1 mg/mL, 0.0564 µmol) or 90 µL of DOTA-IPB-NAPamide stock solution (1mg/mL, 0.0477 µmol), then was heated at 95 °C for 5 min. For the purification of the mixture, solid-phase extraction (SPE) applying a SepPak C18 Plus light cartridge was used based on the same method as above. The quality control of the labeled complexes was carried out with radio-iTLC. For the mobile phase, we applied 0.5 M citric acid (pH 5.5).

#### 3.3.4. Determination of the *logP* Values

After the dilution of the purified radiotracers ([^68^Ga]Ga-DOTA-NAPamide, [^68^Ga]Ga-DOTA-IPB-NAPamide, [^205/206^Bi]Bi-DOTA-NAPamide, [^205/206^Bi]Bi-DOTA-IPB-NAPamide, [^177^Lu]Lu-DOTA-NAPamide, [^177^Lu]Lu-DOTA-IPB-NAPamide) (50 μL, 1–3 MBq) with 450 μL water, 500 μL n-octanol was added to each sample. The mixture was stirred for 3 min, which was followed by centrifugation (9000 rpm) for 5 min. The activity of the samples from n-octanol phase (20 μL) and aqueous phase (20 μL) was analyzed with gamma counter. The procedure was performed in triplicate. For [^68^Ga]Ga-DOTA-NAPamide, [^68^Ga]Ga-DOTA-IPB-NAPamide, [^205/206^Bi]Bi-DOTA-NAPamide, [^205/206^Bi]Bi-DOTA-IPB-NAPamide, [^177^Lu]Lu-DOTA-NAPamide, and for [^177^Lu]Lu-DOTA-IPB-NAPamide the *logP* values were −3.46, −2.12, −3.65, −2.32, −2.6, 1.26, respectively.

#### 3.3.5. Stability Test of [^68^Ga]Ga-DOTA-NAPamide, [^68^Ga]Ga-DOTA-IPB-NAPamide, [^205/206^Bi]Bi-DOTA-NAPamide, [^205/206^Bi]Bi-DOTA-IPB-NAPamide, [^177^Lu]Lu-DOTA-NAPamide, [^177^Lu]Lu-DOTA-IPB-NAPamide

Aqueous solutions (50 µL) of each labeled compound (~1–4 MBq) were added to 50 µL of rat serum at RT. In case of the ^68^Ga- and ^205/206^Bi-labeled radiotracers, the aliquots were taken from the mixture and analyzed at the starting point and 0.5, 1, and 2 h later by radio-iTLC, using the same method as mentioned above. The [^177^Lu]Lu-labeled complexes were analyzed 1, 2, 3, 4 and 24 h later with the same radio-iTLC method (Supplementary Material Figures S5–S10).

#### 3.3.6. EDTA Challenge of [^68^Ga]Ga-DOTA-NAPamide, [^68^Ga]Ga-DOTA-IPB-NAPamide, [^205/206^Bi]Bi-DOTA-NAPamide, [^205/206^Bi]Bi-DOTA-IPB-NAPamide, [^177^Lu]Lu-DOTA-NAPamide, [^177^Lu]Lu-DOTA-IPB-NAPamide

Aqueous solutions (50 µL) of each labeled derivative (~1–4 MBq) were mixed with EDTA aqueous solution (0.2 M, 50 µL, pH 7.4) at RT. Samples from the mixture were examined at certain time points with radio-iTLC, similarly as described above. In the case of ^68^Ga-labeled complexes, the radiochemical purity of the samples was determined by analytical radio-HPLC as described before (Supplementary Material Figure S3).

#### 3.3.7. Metal Challenge of [^68^Ga]Ga-DOTA-NAPamide, [^68^Ga]Ga-DOTA-IPB-NAPamide, [^205/206^Bi]Bi-DOTA-NAPamide, [^205/206^Bi]Bi-DOTA-IPB-NAPamide, [^177^Lu]Lu-DOTA-NAPamide, [^177^Lu]Lu-DOTA-IPB-NAPamide

Aqueous solutions (49 µL) of each radioligand (~1–4 MBq) were mixed with a 1:1 mixture of 0.1 mM ZnCl_2_ and 0.01 mM CuCl_2_ (1 µL), and a 1:1 mixture of 1.02 mM MgCl_2_ and 2.28 mM CaCl_2_ (50 µL) at RT. Aliquots from the mixtures were examined with the above-mentioned radio-iTLC method at the same investigated time points as before. For samples of ^68^Ga-labeled complexes, the above-mentioned analytical radio-HPLC method was used (Supplementary Material Figure S4).

### 3.4. Biology

#### 3.4.1. Experimental Animals

Twelve-week-old C57BL/6 male mice (purchased from Charles River; Animalab Ltd., Budapest, Hungary; *n* = 30) were housed in Individually Ventilated Cages (IVC) maintained at 26 ± 2 °C and 55 ± 10% relative humidity. Laboratory circadian cycle of 12 h was ensured throughout the whole study. Sterile semi-synthetic food (Akronom Ltd., Budapest, Hungary) and water were available ad libitum for the study animals. All experimental mice were housed and kept in accordance with the criteria of the Ethics Committee for Animal Experimentation of the United Kingdom. All animal work was performed under ethics approval (approval number: 16/2020/DEMÁB) obtained from the Ethics Committee for Animal Experimentation of the University of Debrecen.

#### 3.4.2. Cell Lines

MC1-R positive mouse B16F10 melanoma cell lines were purchased from the American Type Culture Collection (CRL-6475™, ATCC, Manassas, VI, USA). Dulbecco’s Modified Eagle’s medium (DMEM, Merck Life Science Ltd., Budapest, Hungary) supplemented with 1% (*v*/*v*) MEM Non Essential Amino Acid solution (Merck Life Science Ltd., Budapest, Hungary), 1% MEM Vitamins solution (Merck Life Science Ltd., Budapest, Hungary), 10% Fetal Bovine Serum (FBS, GIBCO Life Technologies, Billings, MT, USA) and 1% Antibiotic and Antimicotic solution (Merck Life Science Ltd., Budapest, Hungary) was used for the culturing of the B16F10 cells. The cell lines were maintained within a humidified 5% CO_2_ atmosphere at 37 °C. For tumor implantation, we used cells at 85% confluence. The viability of the cell lines was assessed with a trypan blue exclusion test, and it always exceeded 90%.

#### 3.4.3. In Vivo Tumor Models

MC1-R positive melanoma tumors were implanted in C57BL/6 mice (*n* = 30) by the subcutaneous *(sc.)* injection of 1 × 10^5^ B16F10 tumor cells in 100 μL saline into their left shoulder area. 9 ± 1 days post tumor cell implantation, in vivo and ex vivo radiopharmaceutical uptake investigations were performed at an average tumor volume of 124 ± 12 mm^3^.

#### 3.4.4. In Vivo Positron Emission Tomography (PET) Imaging

At 9 ± 1 days post tumor implantation, at an average tumor bulk of approximately 120 mm^3^, the tumor-naive and the B16F10 tumor-bearing mice were intravenously (iv.) injected with 9.36 ± 0.67 MBq of [^68^Ga]Ga-DOTA-NAPamide or [^68^Ga]Ga-DOTA-IPB-NAPamide via the lateral tail vein. After a 90-min-long tracer distribution time, the study mice were subjected to whole-body static PET acquisition (10 min scans at each bed position) under isoflurane-induced anesthesia (AbbVie, Budapest, Hungary; OGYI-T-1414/01) applying a preclinical MiniPET-II small animal PET device (Division of Nuclear Medicine and Translational Imaging, Department of Medical Imaging, Faculty of Medicine, University of Debrecen).

#### 3.4.5. PET Data Assessment

Three-dimensional ordered-subsets expectation-maximization algorithm (3D OSEM) was used for PET image reconstruction. Three-dimensional ellipsoidal volume of interests (VOI) were manually placed around the border of the organ/tissue activity to calculate their radiotracer concentration. The radiopharmaceutical accumulation was presented as standardized uptake values (SUVs). The following formula was applied for the determination of the SUV parameter:

SUV (g/mL) = [VOI activity (Bq/mL)]/[administered activity (Bq)/animal weight (g)]

#### 3.4.6. In Vivo Single-Photon Emission Computed Tomography/Computed Tomography (SPECT/CT) Acquisition

For the evaluation of the tumor-targeting capacity of [^177^Lu]Lu-DOTA-NAPamide and [^177^Lu]Lu-DOTA-IPB-NAPamide, SPECT/CT images were acquired at 24 h postinjection using a nanoSPECT/CT system (Mediso Ltd., Budapest, Hungary). The experimental small animals were *iv.* administered with 6.34 ± 1.07 MBq of [^177^Lu]Lu-DOTA-NAPamide or [^177^Lu]Lu-DOTA-IPB-NAPamide through the lateral tail vein. Followed by a 24 h long uptake period, SPECT data were gathered, and images were reconstructed according to the manufacturer’s instructions applying the Nucline acquisition software and the InterView™ FUSION 1.2 (Mediso Ltd., Budapest, Hungary) image analysis software.

#### 3.4.7. Ex Vivo Organ Distribution Experiments

To accomplish post-imaging organ distribution investigations, 9.36 ± 0.67, 6.34 ± 1.07 and 2.39 ± 0.19 MBq of ^68^Ga-labeled—^177^Lu-labeled—and ^205/206^Bi-labeled NAPamide-based probes were *iv.* Injected, respectively, into the lateral tail vein of both the tumor-naive and the melanoma tumor-carrying experimental mice. Ninety-minutes ([^68^Ga]Ga-DOTA-NAPamide; [^68^Ga]Ga-DOTA-IPB-NAPamide; [^205/206^Bi]Bi-DOTA-NAPamide; [^205/206^Bi]Bi-DOTA-IPB-NAPamide) and 24 h ([^177^Lu]Lu-DOTA-NAPamide; [^177^Lu]Lu-DOTA-IPB-NAPamide) post tracer administration, mice were sacrificed under Forane-induced inhalation anesthesia. Then, the tumor, the blood, and selected organs and tissues were removed, weighed wet and measured for radioactivity applying a calibrated gamma counter (Perkin-Elmer Packard 406 Cobra, Waltham, MA, USA). The counts per minute (CPM) figures for each sample were converted to percent of administered dose per gram of tissue (%ID/g). The uptake values were displayed as the mean %ID/g ± SD.

#### 3.4.8. Statistical Analysis

MedCalc 18.5 commercial software package (MedCalc 18.5, MedCalc Software, Mariakerke, Belgium) was used to perform the statistical analyses. Statistical differences were defined using Student’s two-tailed *t* test, two-way ANOVA and Mann–Whitney U-test. The data are displayed as the mean ± SD, and the significance was set at *p* < 0.05, unless otherwise indicated.

## 4. Conclusions

We successfully synthetized 4-(p-iodophenyl)butanoic acid-modified DOTA-conjugated NAPamide derivative **10** and radiolabeled with ^68^Ga, ^205/206^Bi and ^177^Lu isotopes with high labeling yield. According to the results of the serum stability tests, no significant changes in the stability of the labeled complexes were observed after two hours, moreover in the case of [^177^Lu]Lu-DOTA-IPB-NAPamide even after 24 h.

From the PET image assessment of the ^68^Ga-labeled DOTA-IPB-NAPamide probe, the B16F10 melanoma tumors could be well identified that indicated the target specificity of the radiotracer. Based on the ex vivo biodistribution studies, the integration of the albumin-binding unit led to the enhancement of the tumor uptake and the decrease in renal accumulation of the IPB containing radiotracer, while the circulation time of the modified radiopharmaceutical increased significantly. Furthermore, the ^68^Ga and ^205/206^Bi-labeled DOTA-IPB-NAPamide tracers were encountered in large quantities in the blood and in other off-target organs (e.g., liver) that could be attributable to their slow clearance induced by the introduction of the 4-(p-iodophenyl)butyryl (IPB) group. The obtained results clearly show that in case of the ^68^Ga and ^213^Bi isotopes, the extended biological half-life is not advantageous either in diagnostic or therapeutic applications due to their short half-life.

Considering the enhanced in vivo and ex vivo tumor uptake of the IPB containing ^177^Lu-labeled NAPamide-based radiopharmaceutical even 24 h post injection, prolonged circulation time has a clear diagnostic advantage in the case of labeling with a longer half-life ^177^Lu isotope. However, in addition to the increased biological half-life, the relatively high lipophilicity of the radiotracer resulted in an unexpectedly high background uptake, for example, in the blood, lung, and liver.

Overall, our study confirms that the application of the albumin-binding strategy is a relatively simple and effective method to improve the tumor-targeting potential of peptide-based radiopharmaceuticals labeled with long half-life ^177^Lu isotope. Although the use of an albumin binder with lower affinity—such as p-(tolyl)butyric acid [11]—could improve the tumor-to-background ratios of [^177^Lu]Lu-DOTA-IPB-NAPamide, the optimization of the unexpectedly high non-target uptake of the ^177^Lu-labeled derivative is part of future work.

## Figures and Tables

**Table 1:** Post-imaging biodistribution assessment of [^68^Ga]Ga-DOTA-NAPamide and [^68^Ga]Ga-DOTA-IPB-NAPamide in B16F10 tumor-bearing mice 90 min after intravenous radiopharmaceutical administration and 9 ± 1 days post tumor implantation. %ID/g data are expressed as the mean ± SD. *n* = 5 mice/radiopharmaceutical. Significance level between [^68^Ga]Ga-DOTA-NAPamide and [^68^Ga]Ga-DOTA-IPB-NAPamide accumulation in the selected organs and tissues: *p* ≤ 0.01 (**).

Organ/Tissue	[^68^Ga]Ga-DOTA-NAPamide (%ID/g)	[^68^Ga]Ga-DOTA-IPB-NAPamide (%ID/g)
blood	0.35 ± 0.10	14.21 ± 1.87 **
liver	0.31 ± 0.08	2.64 ± 0.11 **
spleen	0.15 ± 0.02	2.37 ± 0.25 **
kidney	3.07 ± 0.35	4.57 ± 0.88
small intestine	0.35 ± 0.13	2.41 ± 0.03 **
large intestine	0.30 ± 0.01	0.92 ± 1.25
stomach	0.24 ± 0.09	2.97 ± 1.38 **
muscle	0.08 ± 0.02	1.01 ± 0.18 **
lung	0.33 ± 0.06	6.32 ± 0.91 **
heart	0.10 ± 0.06	5.14 ± 0.72 **
brain	0.03 ± 0.01	0.28 ± 0.02 **
fat	0.16 ± 0.03	2.02 ± 0.65 **
**B16-F10 tumor**	**1.18 ± 0.27**	**5.06 ± 1.08** **

SD: standard deviation.

**Table 2:** Ex vivo biodistribution studies of [^205/206^Bi]Bi-DOTA-NAPamide and [^205/206^Bi]Bi-DOTA-IPB-NAPamide in B16F10 tumor-bearing mice 90 min after intravenous radiopharmaceutical application and 9 ± 1 days following B16F10 cell implantation. %ID/g data are displayed as the mean ± SD. *n* = 5 mice/radiopharmaceutical. Significance level between [^205/206^Bi]Bi-DOTA-NAPamide and [^205/206^Bi]Bi-DOTA-IPB-NAPamide accumulation in the selected organs and tissues: *p* ≤ 0.05 (*) and *p* ≤ 0.01 (**).

Organ/Tissue	[^205/206^Bi]Bi-DOTA-NAPamide (%ID/g)	[^205/206^Bi]Bi-DOTA-IPB-NAPamide (%ID/g)
blood	0.34 ± 0.08	13.75 ± 3.58 **
liver	1.09 ± 0.17	2.54 ± 0.91 *
spleen	0.53 ± 0.12	2.07 ± 1.35 **
kidney	8.78 ± 3.61	3.85 ± 0.55
small intestine	0.31 ± 0.19	1.99 ± 0.21 **
large intestine	0.39 ± 0.11	1.02 ± 0.86
stomach	0.18 ± 0.07	1.59 ± 1.11 **
muscle	0.04 ± 0.02	1.20 ± 0.75 **
lung	0.44 ± 0.08	6.14 ± 1.62 **
heart	0.16 ± 0.03	4.52 ± 1.13 **
brain	0.04 ± 0.03	0.25 ± 0.10 **
fat	0.03 ± 0.02	1.50 ± 0.96 **
**B16-F10 tumor**	**3.14 ± 0.32**	**4.50 ± 0.98** *

SD: standard deviation.

**Table 3:** Ex vivo biodistribution studies of [^177^Lu]Lu-DOTA-NAPamide and [^177^Lu]Lu-DOTA-IPB-NAPamide in B16F10 tumor-bearing mice 24 h after intravenous radiotracer administration and 9 ± 1 days post tumor implantation. %ID/g data are expressed as the mean ± SD. *n* = 5 mice/radiopharmaceutical. Significance level between [^177^Lu]Lu-DOTA-NAPamide and [^177^Lu]Lu-DOTA-IPB-NAPamide accumulation in the selected organs and tissues: *p* ≤ 0.01 (**).

Organ/Tissue	[^177^Lu]Lu-DOTA-NAPamide (%ID/g)	[^177^Lu]Lu-DOTA-IPB-NAPamide (%ID/g)
blood	0.02 ± 0.02	3.71 ± 0.73 **
liver	0.24 ± 0.13	1.97 ± 0.13 **
spleen	0.07 ± 0.04	1.32 ± 0.20 **
kidney	1.85 ± 0.79	5.58 ± 1.59
small intestine	0.01 ± 0.01	0.60 ± 0.03 **
large intestine	0.02 ± 0.02	0.61 ± 0.11 **
stomach	0.02 ± 0.01	0.69 ± 0.11 **
muscle	0.00 ± 0.00	0.33 ± 0.07 **
lung	0.04 ± 0.03	2.50 ± 0.24 **
heart	0.01 ± 0.00	1.30 ± 0.12 **
brain	0.00 ± 0.00	0.08 ± 0.01 **
fat	0.01 ± 0.01	0.67 ± 0.34 **
**B16-F10 tumor**	**0.16 ± 0.15**	**4.16 ± 1.86** **

SD: standard deviation.

## Data Availability

Data is contained within the article and supplementary material.

## Supplementary Materials

The following supporting information can be downloaded at: https://www.mdpi.com/article/10.3390/ph16091280/s1, Figure S1: Analytical RP-HPLC chromatograms of compound **10**; Figure S2: Mass spectrum of compound **10**; Figure S3: Stability test of [^68^Ga]Ga-DOTA-IPB-NAPamide in 0.01 M Na_2_EDTA solution; Figure S4: Metal challenge of [^68^Ga]Ga-DOTA-IPB-NAPamide; Figure S5: Radio-iTLC chromatogram of [^68^Ga]GaCl_3_ solution; Figure S6: Stability test of [^68^Ga]Ga-DOTA-IPB-NAPamide in rat serum after 2 h; Figure S7: Radio-TLC chromatogram of [^205/206^Bi]BiCl_3_ solution; Figure S8: Stability test of [^205/206^Bi]Bi-DOTA-IPB-NAPamide in rat serum after 2 h; Figure S9: Radio-TLC chromatogram of [^177^Lu]LuCl_3_ solution; Figure S10: Stability test of [^177^Lu]Lu-DOTA-IPB-NAPamide in rat serum after 2 days.
