# Supplementary material for: Investigation of the Effect on the Albumin Binding Moiety for the Pharmacokinetic Properties of 68Ga-, 205/206Bi-, and 177Lu-Labeled NAPamide-Based Radiopharmaceuticals
Source: Pharmaceuticals (Basel). 2023 Sep 11;16(9):1280. doi: 10.3390/ph16091280 (PMC10536547; doi:10.3390/ph16091280)
Supplement: Supplementary file 1 [file pharmaceuticals-16-01280-s001.zip › pharmaceuticals-2502836-supplementary.pdf]

Supplementary material

Investigation of the effect on the albumin binding moiety for the pharmacokinetic properties of ^{68}Ga-, $^{205/206}$Bi-, and ^{177}Lu-labeled NAPamide-based radiopharmaceuticals

Dániel Szücs[1,2,3], Judit P. Szabó[1,4], Viktória Arató[1,5], Barbara Gyuricza[1], Dezső Szikra[1], Imre Tóth[2], Zita Képes[1], György Trencsényi[1] and Anikó Fekete[1,*]

1 Division of Nuclear Medicine and Translational Imaging, Department of Medical Imaging, Faculty of Medicine, University of Debrecen, Nagyerdei krt. 98., H-4032 Debrecen, Hungary;

2 Department of Physical Chemistry, Faculty of Science and Technology, University of Debrecen, Egyetem tér 1, H-4032 Debrecen, Hungary;

3 Doctoral School of Chemistry, Faculty of Science and Technology, University of Debrecen, Egyetem tér 1., H-4032 Debrecen, Hungary

4 Doctoral School of Clinical Medicine, Faculty of Medicine, University of Debrecen, Nagyerdei krt. 98., H-4032 Debrecen, Hungary

5 Doctoral School of Pharmaceutical Sciences, Faculty of Pharmacy, University of Debrecen, Nagyerdei krt. 98., H-4032 Debrecen, Hungary

* Correspondence: fekete.aniko@science.unideb.hu; Tel.: +36-52-255-510 (ext. 54470)

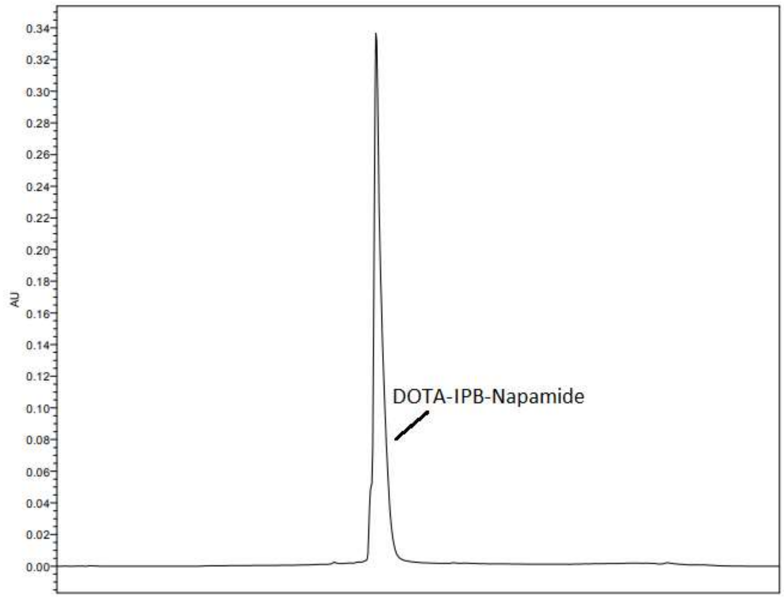

Figure S1 Analytical RP-HPLC chromatograms of compound **10.**

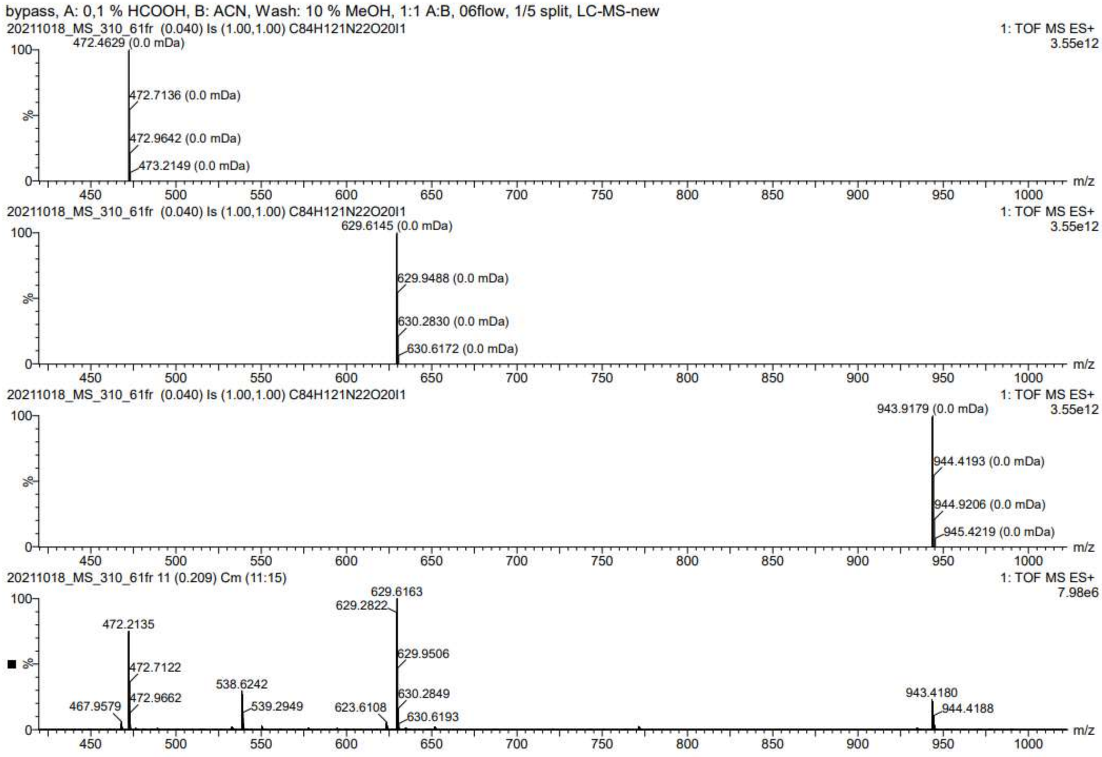

Figure S2 Mass spectrum of compound **10.**

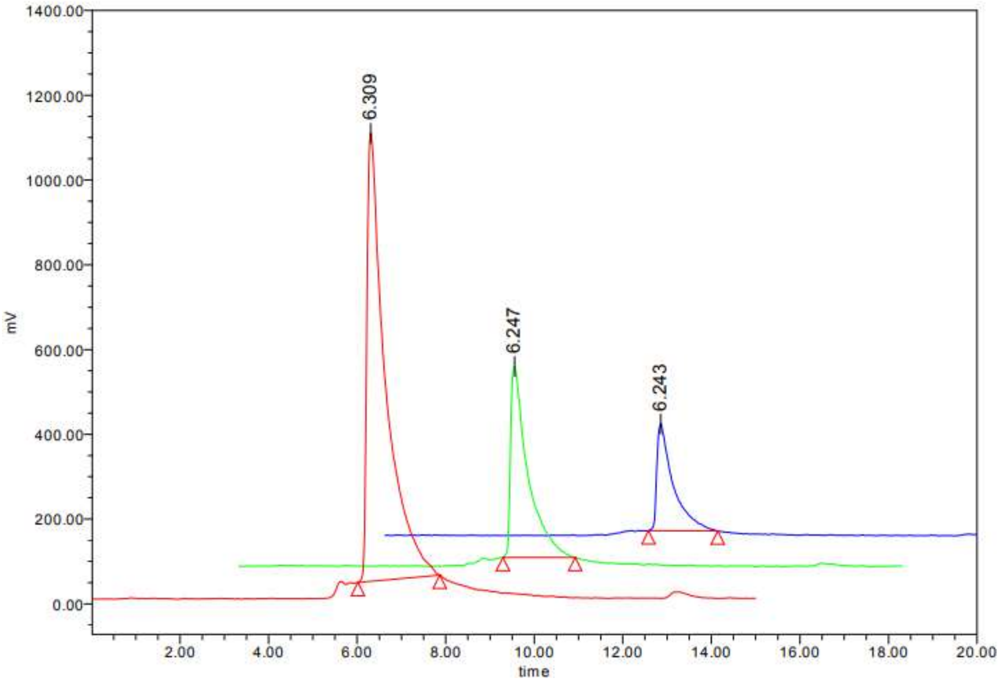

Figure S3 Stability test of [68Ga]Ga-DOTA-IPB-NAPamide in 0.01 M Na_2EDTA solution.

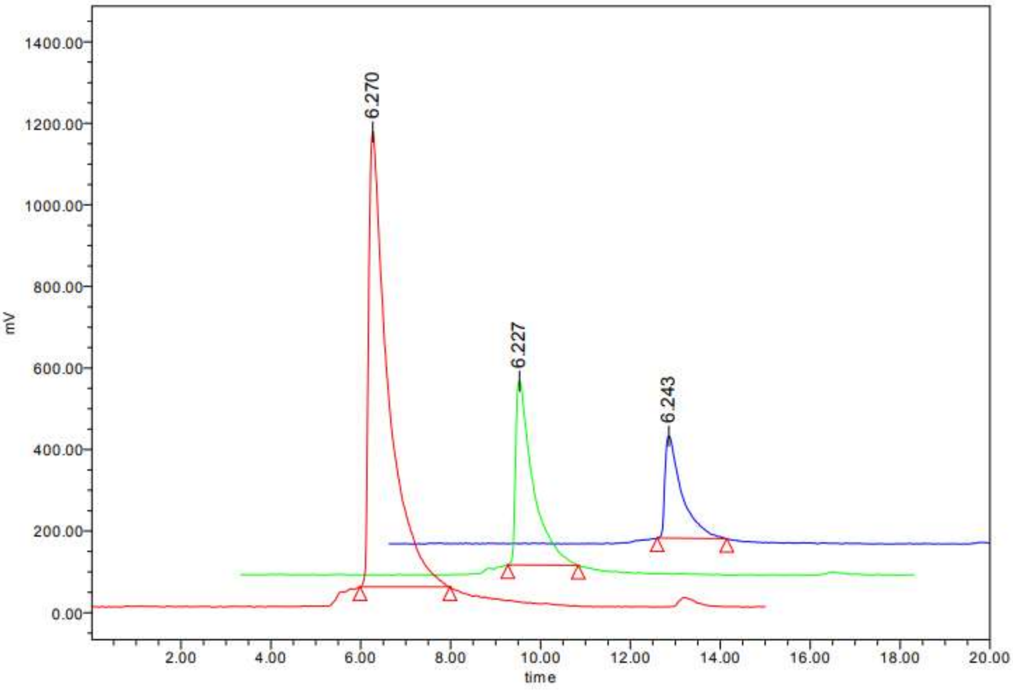

Figure S4 Metal challenge of [68Ga]Ga-DOTA-IPB-NAPamide.

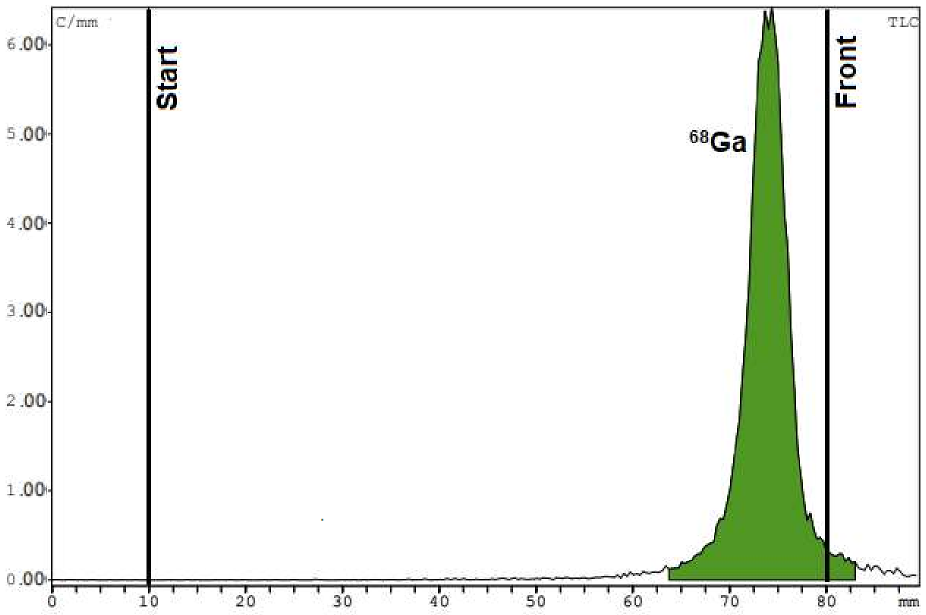

Figure S5 Radio-iTLC chromatogram of [^{68}Ga]GaCl$_3$ solution.

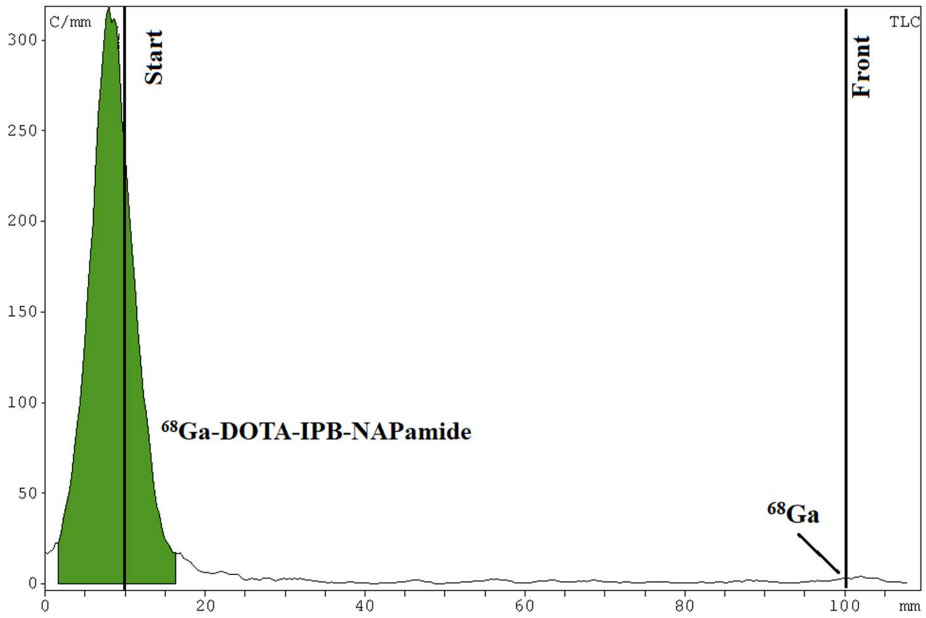

Figure S6 Stability test of [^{68}Ga]Ga-DOTA-IPB-NAPamide in rat serum after 2 hours.

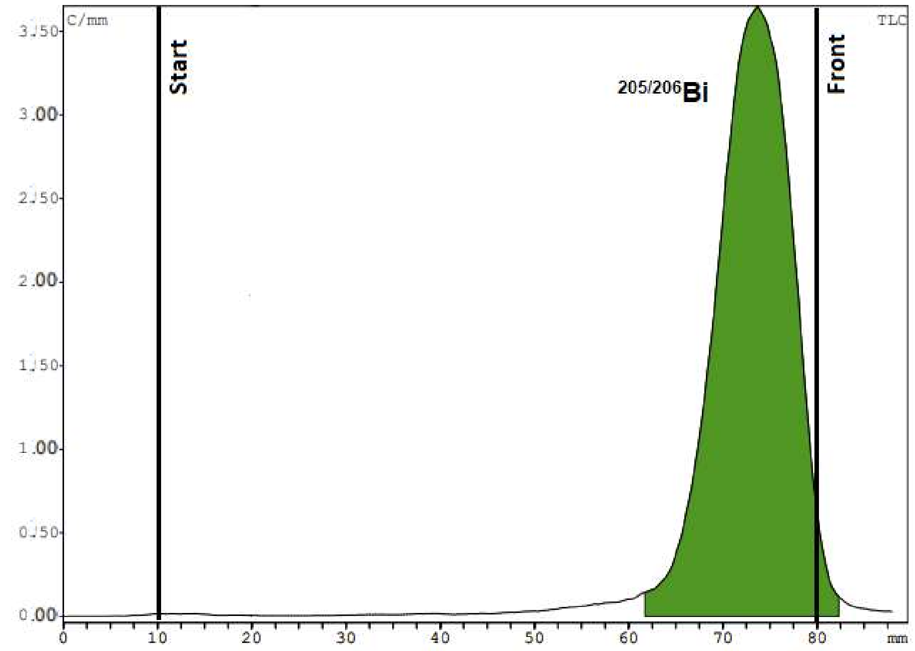

Figure S7 Radio-TLC chromatogram of [$^{205/206}$Bi]$BiCl_3$ solution.

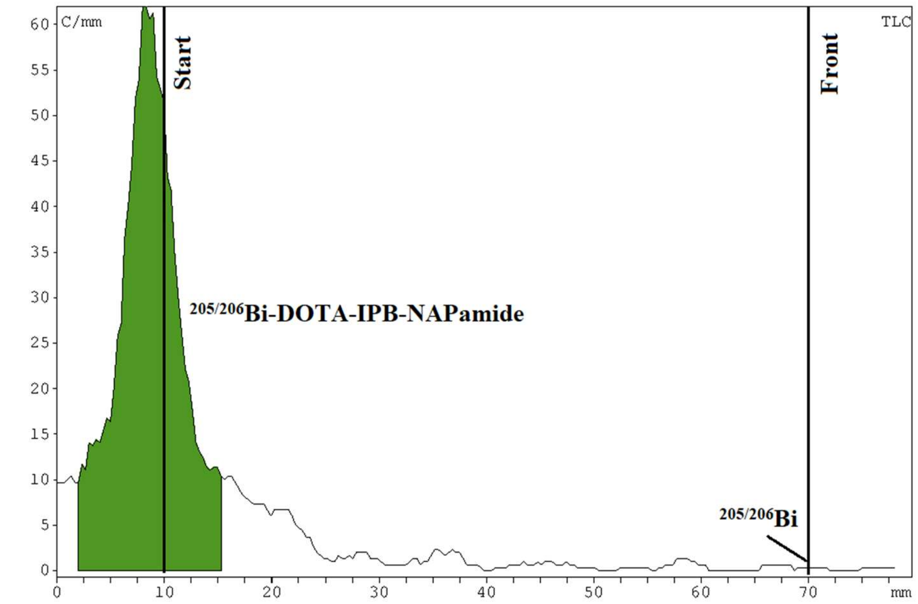

Figure S8 Stability test of [$^{205/206}$Bi]Bi-DOTA-IPB-NAPamide in 0.01 M Na_2EDTA solution after 2 hours.

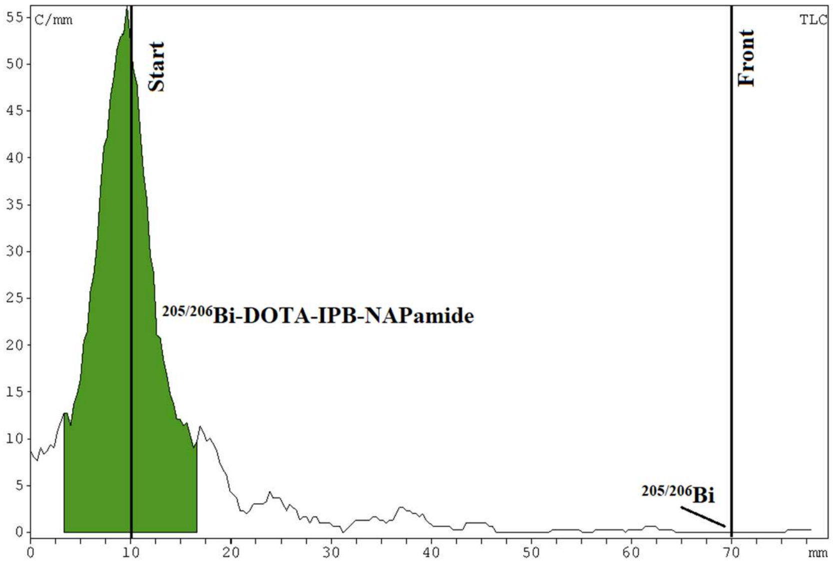

Figure S9 Metal challenge of [$^{205/206}$Bi]Bi-DOTA-IPB-NAPamide after 2 hours.

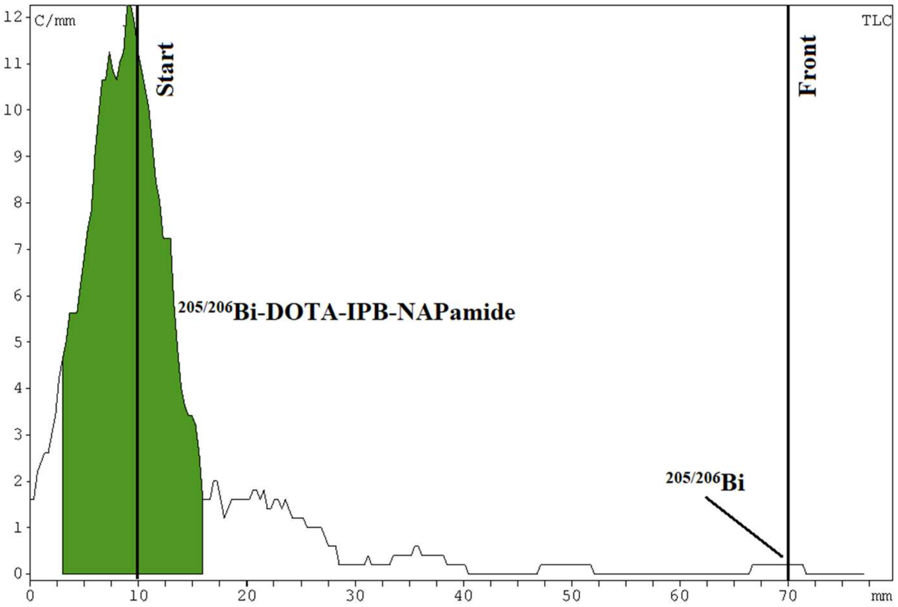

Figure S10 Stability test of [$^{205/206}$Bi]Bi-DOTA-IPB-NAPamide in rat serum after 2 hours.

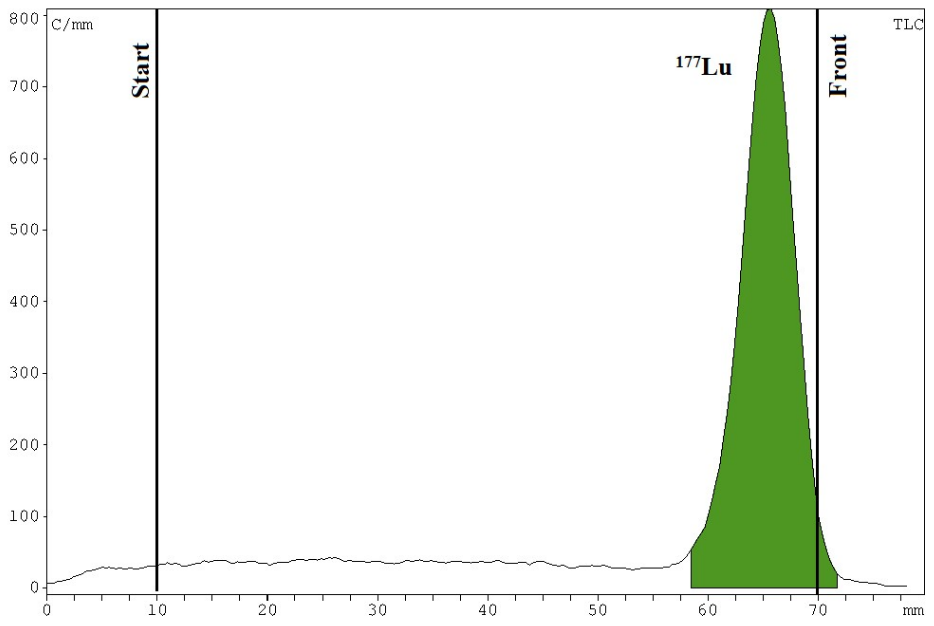

Figure S11 Radio-TLC chromatogram of [^{177}Lu]$LuCl_3$ solution.

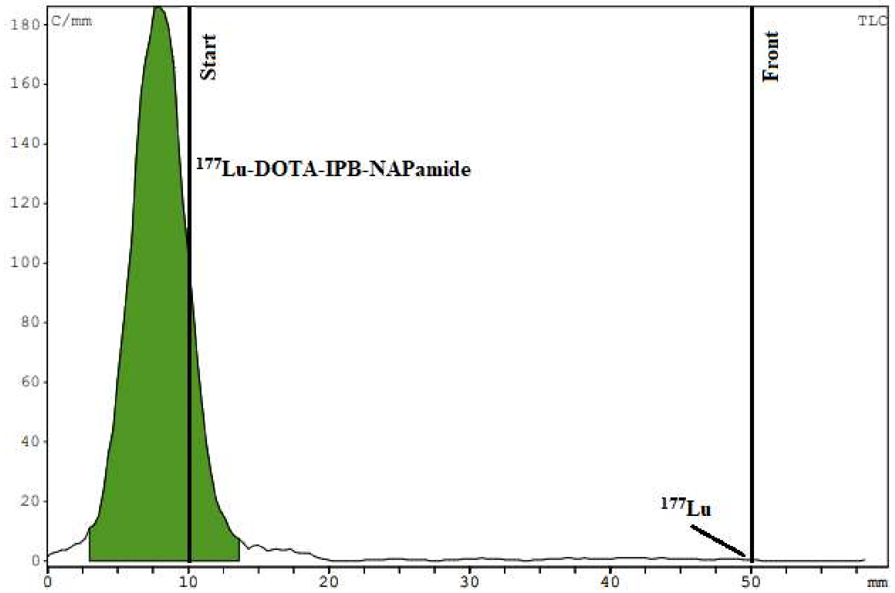

Figure S12 Stability test of [^{177}Lu]Lu-DOTA-IPB-NAPamide in 0.01 M Na_2EDTA solution after 2 days.

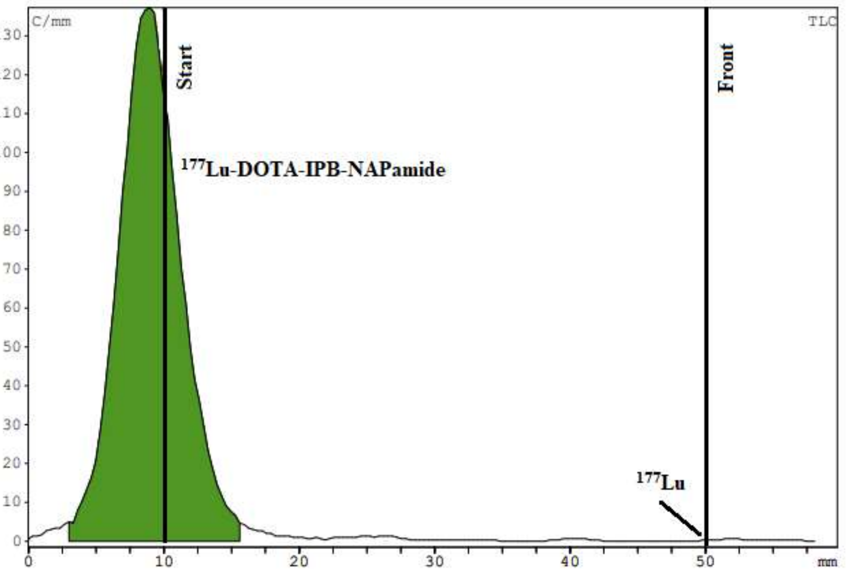

Figure S13 Metal challenge of [^{177}Lu]Lu-DOTA-IPB-NAPamide after 2 days.

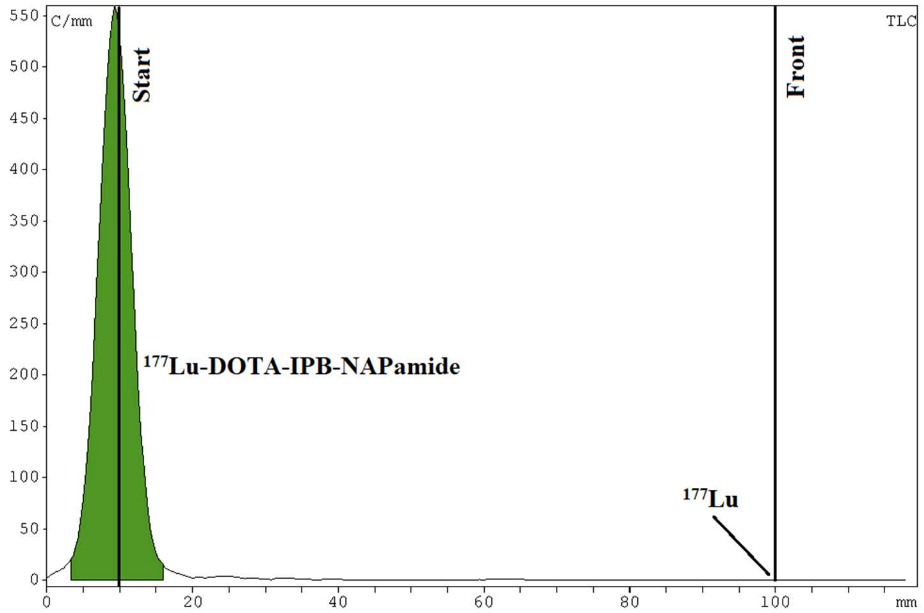

Figure S14 Stability test of [^{177}Lu]Lu-DOTA-IPB-NAPamide in rat serum after 2 days.
